# Influence of Isostatic Pressure on the Elastic and Electronic Properties of K_2_SiF_6_:Mn^4+^

**DOI:** 10.3390/ma15020613

**Published:** 2022-01-14

**Authors:** Mekhrdod Subhoni, Umar Zafari, Chong-Geng Ma, Alok M. Srivastava, William W. Beers, William E. Cohen, Mikhail G. Brik, Michal Piasecki, Tomoyuki Yamamoto

**Affiliations:** 1 College of Sciences & CQUPT-BUL Innovation Institute, Chongqing University of Posts and Telecommunications, Chongqing 400065, China; cgma.ustc@gmail.com; 2 Kagami Memorial Research Institute for Materials Science and Technology, Waseda University, Tokyo 169-0051, Japan; tymmt@waseda.jp; 3 Center of Innovative Development of Science and New Technologies, National Academy of Sciences of Tajikistan, Dushanbe 734025, Tajikistan; zafari_umar@mail.ru; 4 Physical Technical Institute, National Academy of Sciences of Tajikistan, Dushanbe 734063, Tajikistan; 5 Current Lighting Solutions LLC, 1099 Ivanhoe Road, Cleveland, OH 44110, USA; srivastaam@outlook.com (A.M.S.); william.beers@gecurrent.com (W.W.B.); bill.cohen@gecurrent.com (W.E.C.); 6 Institute of Physics, University of Tartu, W. Ostwald Str. 1, 50411 Tartu, Estonia; 7 Faculty of Science and Technology, Jan Długosz University, Armii Krajowej 13/15, PL-42200 Częstochowa, Poland; m.piasecki@ujd.edu.pl; 8 Academy of Romanian Scientists, Ilfov Str. No. 3, 050044 Bucharest, Romania; 9 Inorganic Chemistry Department, Uzhhorod National University, Pidhirna Str. 46, 88000 Uzhhorod, Ukraine; 10 Faculty of Science and Engineering, Waseda University, Tokyo 169-8555, Japan; 11 Institute of Condensed-Matter Science, Waseda University, Tokyo 169-8555, Japan

**Keywords:** K_2_SiF_6_, Mn^4+^, isostatic pressure, interionic distances, bulk modulus, elastic constant, Debye temperature, crystal field strength 10*Dq*, emission energy

## Abstract

Isostatic pressure effects on the elastic and electronic properties of non-doped and Mn^4+^-doped K_2_SiF_6_ (KSF) have been investigated by first-principles calculations within density functional theory (DFT). Bulk modulus was obtained by the Murnaghan’s equation of states (EOS) using the relationship between volume and pressures at pressures between 0 and 40 GPa, and elastic constants were calculated by the stress–strain relationship giving small distortions at each pressure point. The other elastic parameters such as shear modulus, sound velocity and Debye temperature, which can be obtained from the elastic constants, were also estimated. The influence of external isostatic pressure on the electronic properties, such as crystal field strength 10*Dq* and emission energy of ^2^E → ^4^A^2^ transition (*E_em_*), of KSF:Mn^4+^ was also studied. The results suggest that 10*Dq* and *E_em_* linearly increase and decrease, respectively, with increasing pressure.

## 1. Introduction

Mn^4+^-doped phosphors as red photon generating systems have been extensively studied over the past decades due to their great promise for use in LED devices [1,2,3,4,5,6,7]. Among a large number of Mn^4+^ activated red-emitting phosphors, K_2_SiF_6_:Mn^4+^ (KSF:Mn^4+^) has been developed as generators of red photons in phosphor-converted white LEDs (pc-LEDs). The sharp-line emission of KSF:Mn^4+^ peaks at about 630 nm, where the human eye sensitivity to red light is still quite high. With minimum emission beyond 650 nm, the emission spectrum of KSF:Mn^4+^ is suitable for supplying the red photons necessary to produce white light with high efficacy (lumens per watt) and color rendering index (CRI) in pc-LEDs. Given its commercial importance, detailed analyses of the spectroscopic properties, such as absorption and emission spectra, electronic and geometric properties of KSF:Mn^4+^ were performed [8,9,10] and recently this red phosphor was commercialized [11].

The optical properties of the Mn^4+^ ions (with the 3d^3^ electron configuration) are strongly influenced by the composition and crystal structure of the host lattice. In the Tanabe–Sugano diagram for the d^3^ electron configuration, the “Mn^4+^-ligand” bonding covalence defines the emission energy of the ^2^E → ^4^A_2_ spin-forbidden transition and the strength of the octahedral crystal field denoted as 10*Dq*. It was determined by DFT calculations that weak Mn^4+^–ligand hybridization generally leads to higher Mn^4+^ emission energies [12]. The hybridization between the Mn^4+^ ions and ligands is related to the Mn^4+^–ligand distances. The application of pressure is one of the state parameters that can change the peak energy and 10*Dq* by reducing the inter-atomic distances. Studies of the Mn^4+^ optical properties under external pressure have been reported in the archival literature [13,14,15]. The goal of this study was to quantitatively explore by first-principles calculations within density functional theory, the electronic, elastic and optical properties of K_2_SiF_6_ in the absence and presence of the activator ion (Mn^4+^).

## 2. Computational Method

All the density functional calculations in this paper were performed by the plane-wave basis projector augmented wave package, VASP [16], using the generalized gradient approximation proposed by Perdew, Burke and Ernzerhof (GGA-PBE) [17] to express electron–electron correlation. The space group of KSF is Fm-3m with a lattice constant of 8.134 Å [18], in which the Si^4+^ ions are surrounded by six F^−^ ions and the local site symmetry is described by the O_h_ point group. The Mn^4+^-doped KSF models were constructed by replacing one Si^4+^ ion by one Mn^4+^ ion in the unit cell of KSF, which includes 36 atoms corresponding to four formula units. After careful convergence tests with respect to the plane wave cut-off energy, 900 eV was selected for the energy cut-off in all the calculations. The Monkhorst–Pack k-points grid sampling [19] was set as 4 × 4 × 4. The structural parameters of non-doped KSF and KSF:Mn^4+^ were optimized by allowing relaxations of the lattice constants and internal atomic positions under isostatic external pressures between 0 and 40 GPa by a 5 GPa step.

## 3. Results and Discussion

The calculated and experimental lattice constants of non-doped KSF and KSF:Mn^4+^ are summarized in Table 1 together with the calculated Si-F and Mn-F bond lengths. The experimental lattice constant of non-doped KSF, *a* = 8.134 Å [18], is reproduced well by the current calculations, *a* = 8.336 Å, within a typical overestimation due to the GGA-PBE functional use. It is noted here that a slight volume expansion occurs after inclusion of the Mn^4+^ ions into KSF host. The calculated lattice constant of KSF:Mn^4+^ is 8.357 Å, which is larger than that of KSF 8.336 Å. This expansion can be simply explained by the difference in ionic radii of Si^4+^ (0.40 Å) and Mn^4+^ (0.53 Å). The calculated Mn^4+^–F^–^ bond-length is also larger than the calculated Si^4+^–F^–^ bond-length by 6.69%, which supports the earlier experimental result of +7.43% [20].

**Figure 1:**
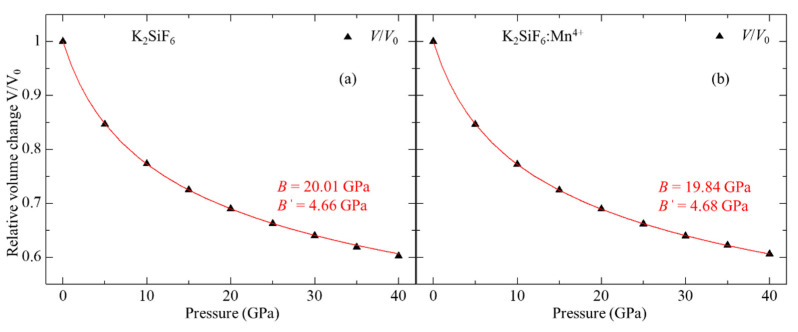
Calculated relative volume *V*/*V*_0_ of (**a**) non-doped and (**b**) Mn^4+^-doped K_2_SiF_6_ as a function of pressure. Red solid curves denote fitting results to the Murnaghan’s equations of state.

The calculated pressure dependence of the relative volume change *V*/*V*_0_ for non-doped KSF is plotted in Figure 1a, which is fitted to the Murnaghan’s equation of state (EOS) [21] expressed by:

(1) V/V0=(1+PB′B)−1B′

where *V* and *V*_0_ are volumes at pressure *P* and ambient pressure, respectively, and *B* and *B*’ are bulk modulus and its pressure derivative, respectively. This fitting yields that *B* and *B*’ are 20.01 GPa and 4.68, respectively. Our results agree well with the earlier study [22] using GGA-PBE, *B* = 21.79 GPa and *B*’ = 4.47, although a slight difference appears between these two from a difference in calculating methods, i.e., projector augmented wave and pseudopotential methods in the current and earlier [22] studies, respectively. To the best of our knowledge, no experimental bulk modulus for KSF has been reported yet. For Mn^4+^-doped KSF, *V*/*V*_0_ is also plotted in Figure 1b, which is also fitted to the Murnaghan’s EOS. The fitting yields *B* = 19.84 GPa and *B*’ = 4.68, which implies that inclusion of Mn^4+^ into KSF leads to a decrease of the bulk modulus.

**Figure 2:**
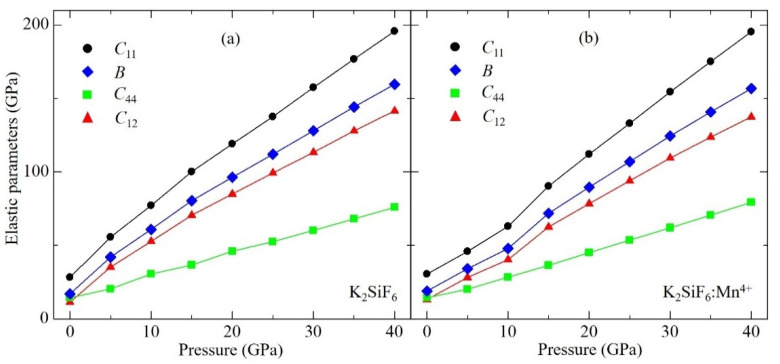
Calculated elastic constants *C_ij_* and bulk moduli B for (**a**) non-doped and (**b**) Mn^4+^-doped K_2_SiF_6_ as a function of pressure.

**Figure 3:**
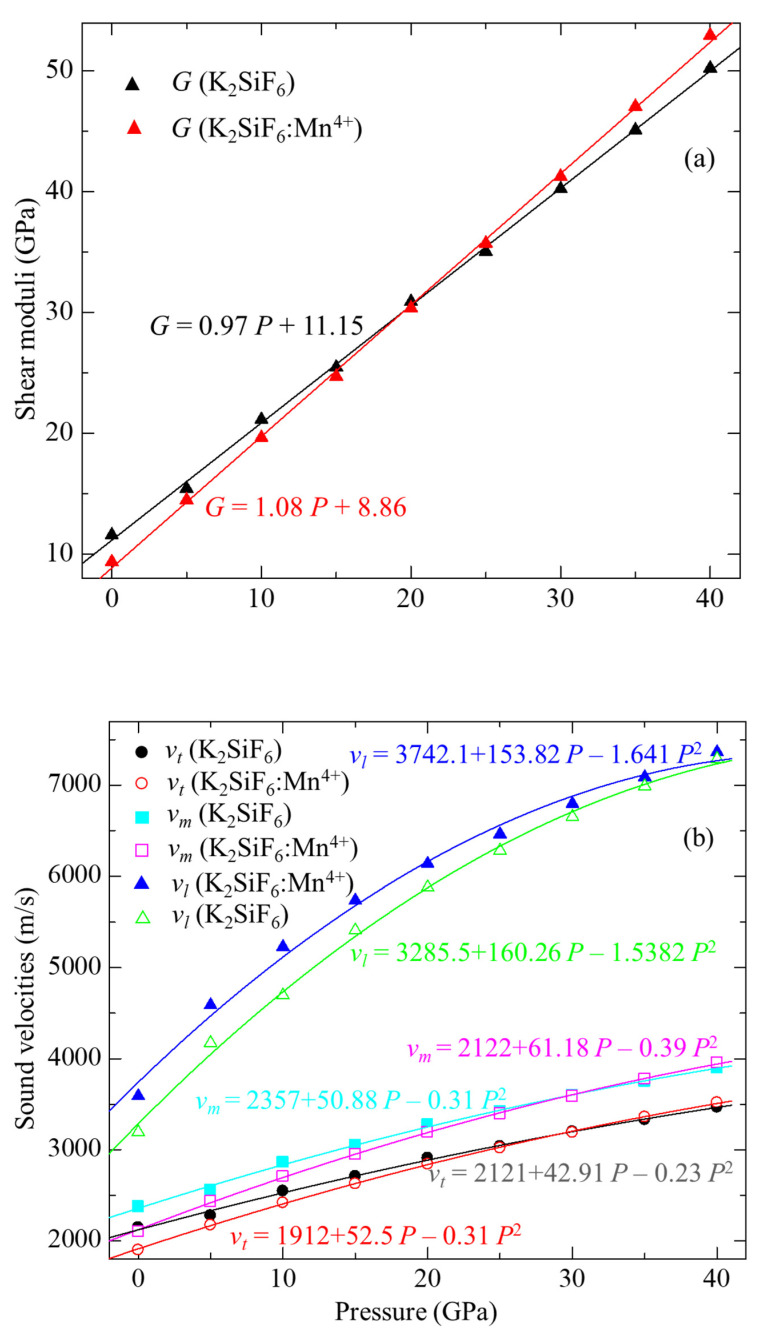

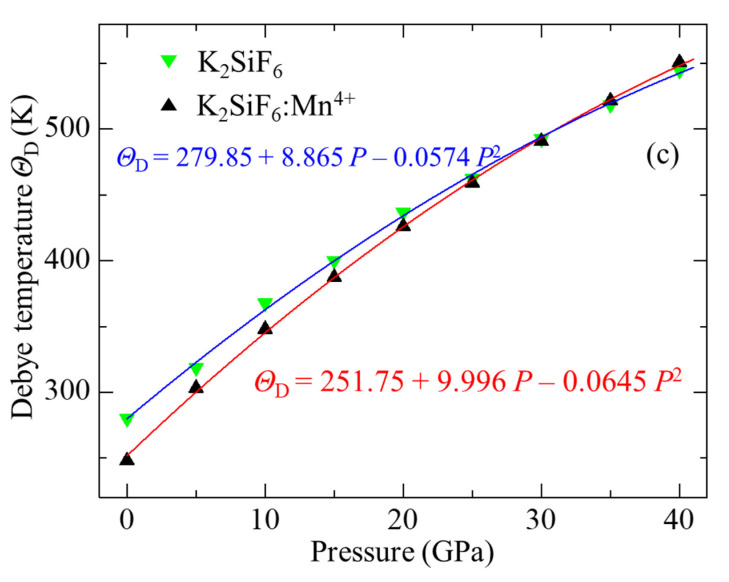
Calculated (**a**) shear moduli, (**b**) sound velocities and (**c**) Debye temperatures for the non-doped and Mn^4+^-doped K_2_SiF_6_ as a function of pressure.

The elastic constants of non-doped and Mn^4+^-doped KSF were calculated using a stress-strain method [23] implemented in VASP, which are summarized in Table 2 and plotted in Figure 2 as a function of pressure. Here the optimized structure at each pressure was used to calculate the elastic constants. Three irreducible elastic constants for the cubic structure, i.e., *C*_11_, *C*_12_ and *C*_44_, at zero-pressure were reported in the earlier study [22]; the values are 31.90, 9.28 and 15.10 GPa, respectively. Those in the current study show similar values of 28.2, 11.3 and 14.3 GPa for *C*_11_, *C*_12_ and *C*_44_, respectively. The difference between these two studies is derived from the difference in calculating conditions as discussed above for the bulk modulus. The calculated *C*_11_ and *C*_12_ values for the non-doped KSF are larger than those of KSF:Mn^4+^ at all pressures calculated in this work. On the other hand, the calculated *C*_44_ of non-doped KSF is larger than that of KSF:Mn^4+^ at zero pressure, the difference between these two becoming smaller as pressure increases, and finally *C*_44_ of KSF:Mn^4+^ becomes larger than that of non-doped KSF between 20 and 25 GPa. It can be noted that all the elastic constants of both non-doped and KSF:Mn^4+^ increase with increase of pressure, in which *C*_11_ and *C*_12_ increase more rapidly than *C*_44_ as shown in Figure 3.

Using these calculated elastic constants, sound velocities can be obtained. The mean sound velocity *v_m_* is expressed [24] in terms of the longitudinal sound velocity *v_l_* and the transverse one *v_t_* as:

(2) $$v_{m}={\left[\frac{1}{3}\left(\frac{2}{v_{t}^{3}}+\frac{1}{v_{l}^{3}}\right)\right]}^{-1/3}$$

in which *v_l_* and *v_t_* are calculated [25] by:

(3) $$v_{l}=\sqrt{\frac{3B+4G}{3\rho }}\;\mathrm{and}\;v_{t}=\sqrt{\frac{G}{\rho }}$$

Here $G=\left(G_{V}+G_{R}\right)/2$ is the isotropic shear modulus, in which $G_{V}=\left(C_{11}-C_{12}+3C_{44}\right)/5$ is the Voight’s shear modulus (an upper limit for *G*), and $G_{R}=5\left(C_{11}-C_{12}\right)C_{44}/\left[4C_{44}+3\left(C_{11}-C_{12}\right)\right]$ is the Reuss’s shear modulus (a lower limit for *G*). Debye temperature, *Θ*_D_, is calculated using the following equation [25]:

(4) $$\Theta_{D}=\frac{h}{k}{\left[\frac{3n}{4\pi }\left(\frac{N_{A}\rho }{M}\right)\right]}^{1/3}v_{m}$$

where *h* and *k* are the Planck’s and Boltzmann’s constants, respectively, *N_A_* is the Avogadro’s number, *ρ* is the density, *M* is the molecular weight, and *n* denotes the number of atoms per formula unit (here nine for KSF). The calculated shear modulus, *G*, sound velocities, *v_m_*, *v_t_* and *v_l_*, and Debye temperature, *Θ*_D_, as a function of pressure for both non-doped and Mn^4+^-doped KSF are summarized in Table 3 and plotted in Figure 3a–c for shear moduli, sound velocities, and Debye temperature, respectively. The *G*, *v_m_*, *v_t_*, *v_l_* and *Θ*_D_ values for both systems increase with pressure. Inclusion of Mn^4+^ into KSF leads to a decrease of *G*, *v_m_*, *v_t_*, *v_l_* and *Θ*_D_ at zero pressure. Each of the differences in those parameters between non-doped and Mn^4+^-doped KSF becomes smaller with increase of pressure. Calculated *G* of KSF:Mn^4+^ becomes larger than that of non-doped KSF at pressures between 20 and 25 GPa as in the case of *C*_44_, while *v_m_*, *v_l_* and *Θ*_D_ of KSF:Mn^4+^ becomes larger than that of the non-doped one between 30 and 35 GPa.

**Figure 4:**
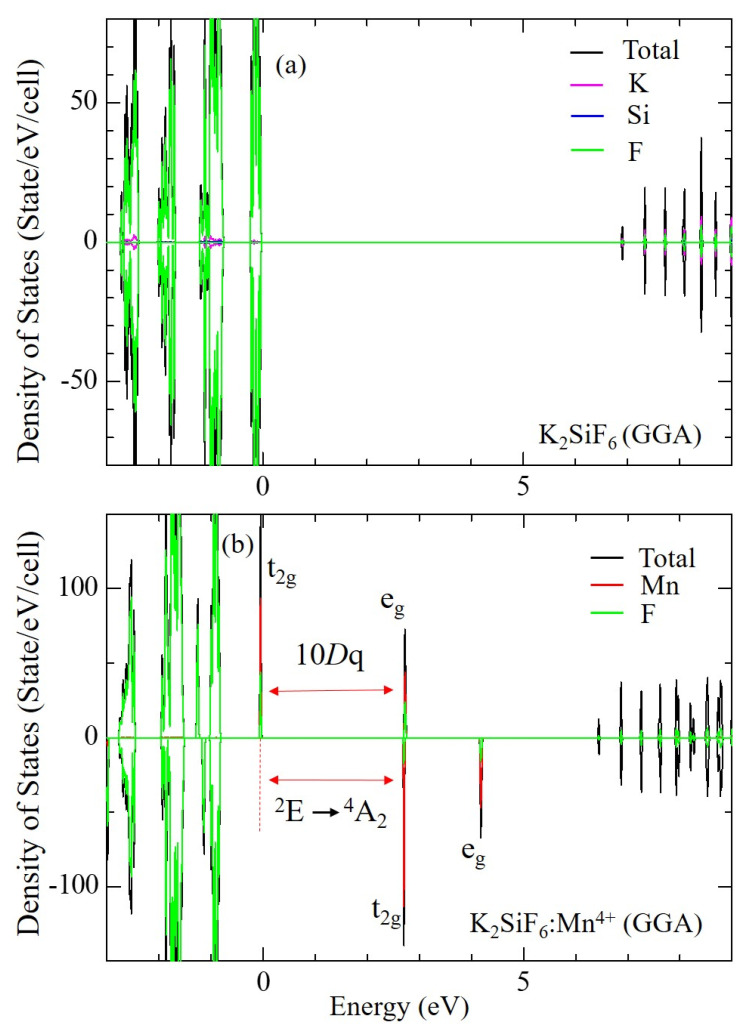
Calculated electronic density of states of (**a**) non-doped and (**b**) Mn^4+^-doped K_2_SiF_6_.

Calculated electronic densities of states (DOSs) of non-doped and Mn^4+^-doped KSF at 0 GPa are shown in Figure 4. As illustrated in this figure, new orbitals associated with the Mn^4+^ 3d orbitals appear in the band gap due to a doping of Mn^4+^ ion. Current results are consistent with our previous report [26], although the previous one was undertaken with larger super cells expanded by 2 × 1 × 1. The Mn^4+^ ion in KSF is surrounded by six F^−^ ions in a crystal environment of cubic symmetry. Hence, the 3d ground-state splits into the triply and doubly degenerated t_2g_ and e_g_ orbitals, respectively. The3d states of Mn^4+^ hybridize with the F^−^ 2p states in both t_2g_ and e_g_ orbitals as shown in Figure 4b. The Mn^4+^ ion has three 3d electrons, which fully occupy t_2g_ up-spin state.

**Figure 5:**
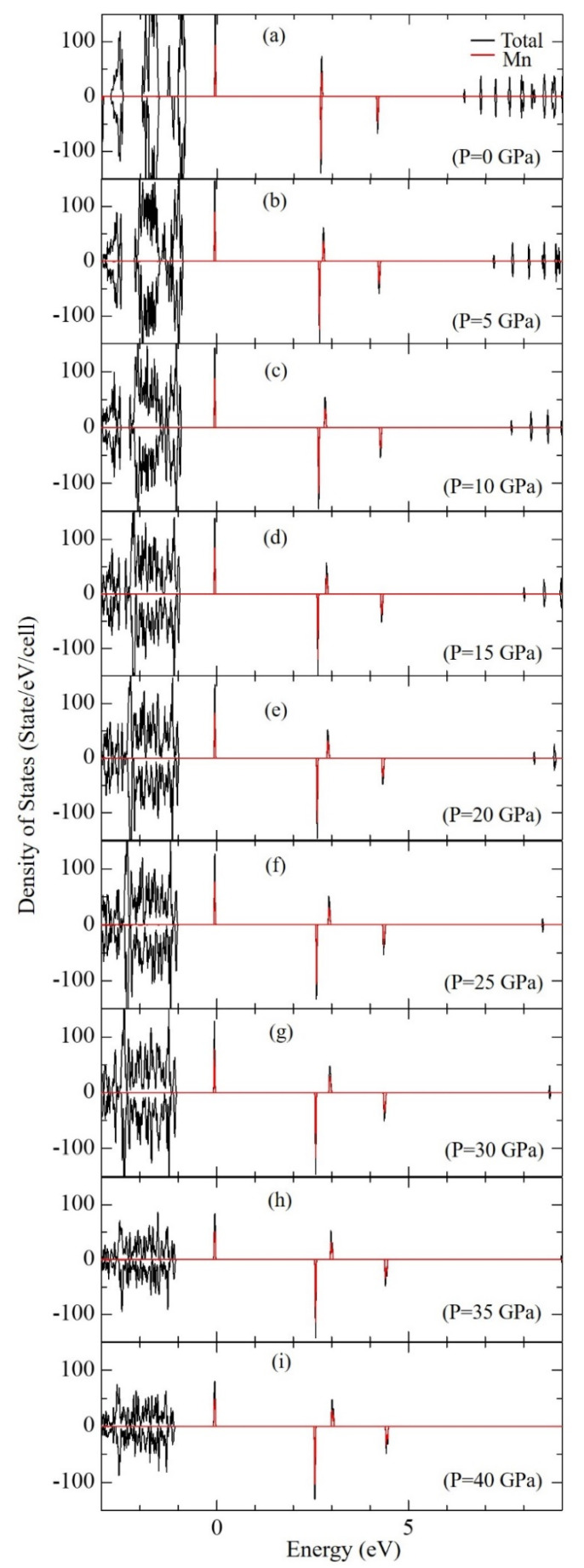
Calculated densities of states (DOSs) of Mn^4+^-doped K_2_SiF_6_ at P = (**a**) 0, (**b**) 5, (**c**) 10, (**d**) 15, (**e**) 20, (**f**) 25, (**g**) 30, (**h**) 35 and (**i**) 40 GPa, respectively.

**Figure 6:**
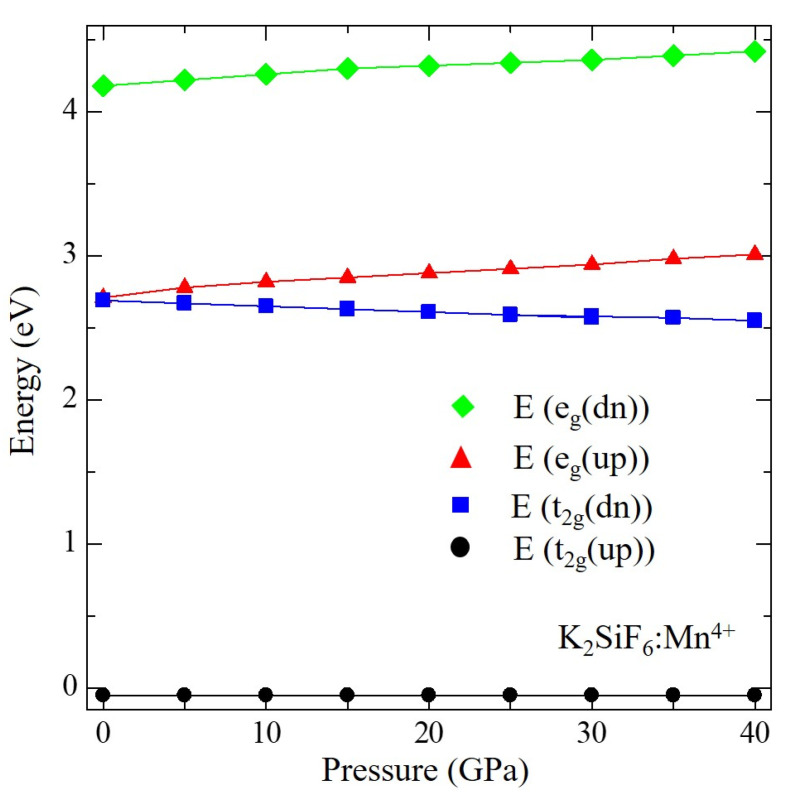
Calculated energy levels of the t_2g_-up, t_2g_-down, e_g_-up and e_g_-down states of Mn^4+^-doped K_2_SiF_6_ as a function of pressure.

**Figure 7:**
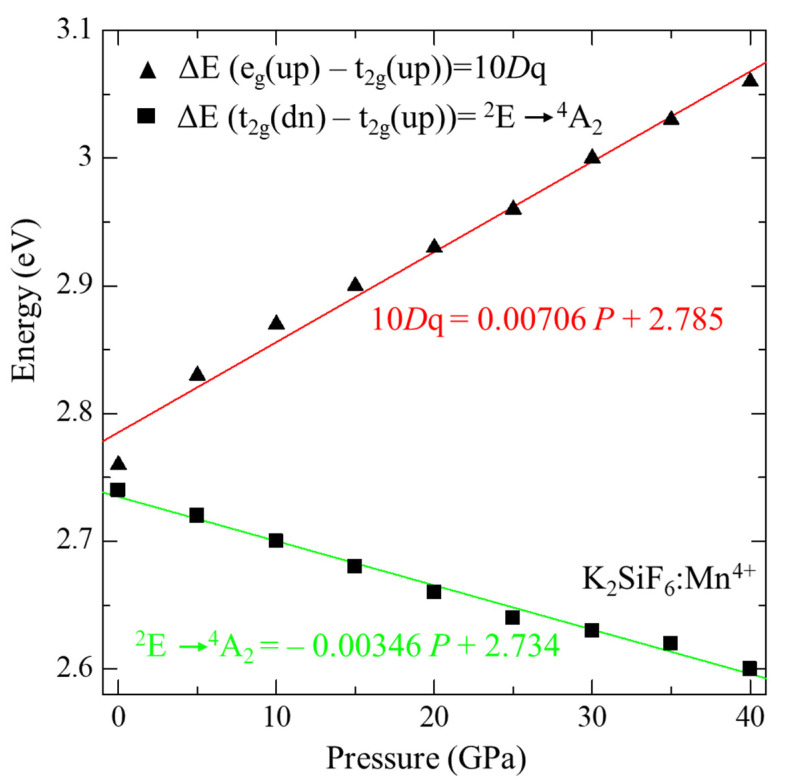
Calculated emission energy, *E_em_*, and 10*Dq* for Mn^4+^-doped K_2_SiF_6_ as a function of pressure. The solid lines are the least-square linear fittings.

**Figure 8:**
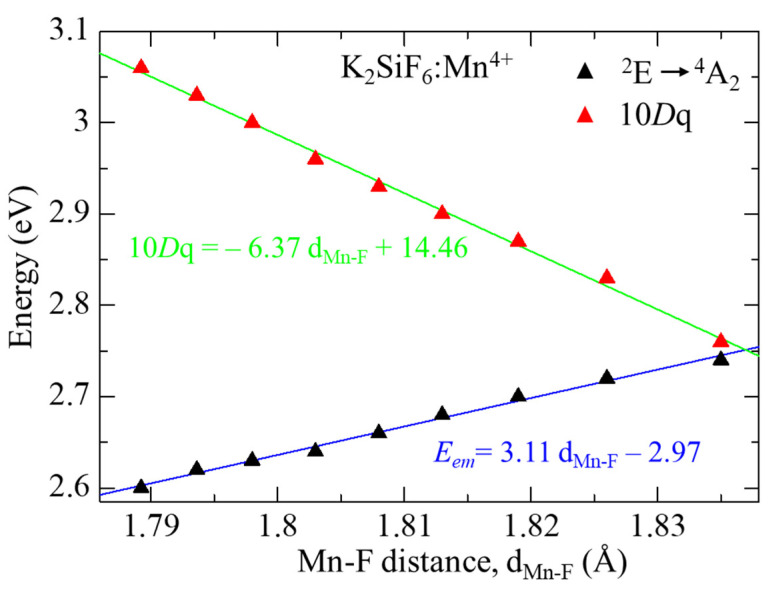
Calculated 10*Dq* parameter and *E_em_* for K_2_SiF_6_:Mn^4+^ as a function of Mn-F bond length.

To discuss the influence of pressure on the electronic structure, calculated DOSs of KSF:Mn^4+^ are compared among those at different pressures between 0 and 40 GPa, which are shown in Figure 5. The energies of the top of each state originating from 3d orbital of Mn^4+^ ion, i.e., up-spin and down-spin states of t_2g_ and e_g_, are plotted in Figure 6. As the top of the occupied band was set to zero in Figure 5, the tops of all t_2g_ up-spin states were located at zero. On the other hand, down-spin t_2g_ states shift to the lower energy side, while up- and down-spin e_g_ states shift to the higher energy side with increasing pressure. Calculated 10*Dq*, which denotes the crystal field strength and is defined as a difference in energy between up-spin t_2g_ and e_g_ states, at different pressures is summarized in Table 4 and plotted in Figure 7. Calculated 10*Dq* value at zero pressure of 2.76 eV agrees well with the experimental value of 2.74 eV [27], although our current calculations were carried out by GGA-PBE without the effect of strong electron correlation for Mn 3d electrons. As shown in these tables and figures, 10*Dq* increases linearly as pressure increases. Least square fitting to the pressure dependence of 10*Dq* yields 10*Dq* = 0.00346 *P* + 2.734. In addition, from a geometrical point of view, 10*Dq* values are plotted in Figure 8 as a function of bond length between Mn^4+^ and sounding F^−^ ions. As shown in this figure, a very good linear relation can be found between 10*Dq* and Mn-F bond length. The least-square fitting result is 10*Dq* = −6.37 d_Mn-F_ + 14.46. This result implies that 10*Dq* can be estimated by this equation for other Mn^4+^-doped fluorides, in which the doped Mn^4+^ ion is located at the O_h_ symmetry point indicated by six F^−^ ions. This result is consistent with our previous study on A_2_SiF_6_ [26], where A = K, Rb and Cs, in which 10*Dq* decreases with increasing Mn^4+^−F^−^ bond-length.

The red emission from Mn^4+^ ion is assigned to the transition from the first excited state ^2^E to the ground state ^4^A_2_ of the Mn^4+^ 3d state. Electron configuration of the ^4^A_2_ state of the Mn^4+^ ion is all three electrons on up-spin t_2g_ while that of ^2^E is two electrons on up-spin t_2g_ and one on down-spin t_2g_ states. Here, the transition energy from ^2^E to ^4^A_2_, *E_em_*, is calculated by an energy gap between the up-spin and down-spin t_2g_ states within a one electron approximation to avoid complicated calculations considering multielectron effects to obtain total electronic energy difference between the ^2^E and ^4^A_2_ states. The calculated *E_em_* value at zero pressure is 2.74 eV, which is larger than the experimental value of 1.99 eV [9]. This overestimation is derived from the method to obtain *E_em_*. However, the change in *E_em_* can be discussed qualitatively as in our earlier report on KSF:Mn^4+^ [26]. As the d and f electrons are localized, consideration of a strong electron–electron correlation effect, such as the DFT + U method we have undertaken previously [26], may lead to a better reproduction of *E_em_* than the current calculations with GGA-PBE. In addition, the calculated *E_em_* at zero pressure, i.e., 2.74 eV, is larger than that in the previous study on KSF:Mn^4+^ [26]. Here, a 1 × 1 × 1 unit cell was used, whereas a 2 × 1 × 1 super cell was employed in our previous study [26], which yield a difference in calculated *E_em_* at zero pressure. *E_em_* as a function of pressure is summarized in Table 4 and plotted in Figure 7. Contrary to the change in 10*Dq*, *E_em_* decreases almost linearly, *E_em_* = −0.00346 *P* + 2.734, with increasing pressure. The calculated *E_em_* as a function of Mn^4+^–F^−^ bond length is also plotted in Figure 8 for KSF:Mn^4+^. It can be seen that the ^2^E → ^4^A_2_ transition energy has very good linear relation with Mn–F bond length, *E_em_* = 3.11 d_Mn-F_ – 2.97.

## 4. Conclusions

First-principles calculations have been carried out for the non-doped and Mn^4+^-doped K_2_SiF_6_ to study the influence of isostatic pressure on the geometric structure, elastic and electronic properties of K_2_SiF_6_:Mn^4+^ red phosphor. In particular, the pressure effect on the elastic properties such as elastic constants, shear modulus, sound velocity and Debye temperature between non-doped and Mn^4+^-doped systems was discussed. Pressure dependence of two important electronic parameters, i.e., 10*Dq* and the emission energy of the ^2^E → ^4^A_2_ transition, of KSF:Mn^4+^ have been investigated, which yield good linear relationships both between 10*Dq* and external pressure and between emission energy of ^2^E → ^4^A_2_ and pressure. It can be noted that the current analysis of the pressure effect on 10*Dq* and emission energy of the ^2^E → ^4^A_2_ transition provided empirical formulae to obtain these values as a function of bond-length between Mn^4+^ and surrounding F^−^ ions, which can be used for the estimation of these important parameters for other Mn-doped fluorides.

## Figures and Tables

**Table 1:** Comparison of lattice constants, *a*, and bond lengths of Si–F and Mn–F in the non-doped and Mn^4+^-doped K_2_SiF_6_, respectively, between experiments and calculations.

System		Calc. (Å)	Exp. (Å)
K_2_SiF_6_	*a*	8.336	8.134 ^a^
	Si-F	1.720	1.683 ^a^
K_2_SiF_6_:Mn^4+^	*a*	8.357	
	Mn-F	1.835	1.807 ^b^

^a^ Ref. [18]. ^b^ Ref. [20].

**Table 2:** Pressure dependence of the elastic constants *C_ij_* (all in GPa) for the non-doped and Mn^4+^- doped K_2_SiF_6_.

System	Pressure	*C*_11_	*C*_12_	*C*_44_
K_2_SiF_6_	0	28.2	11.3	14.3
	5	55.6	35.2	20.4
	10	77.1	52.7	30.5
	15	100.1	70.5	36.6
	20	119.0	84.9	46.0
	25	137.5	99.2	52.5
	30	157.4	113.3	60.1
	35	176.7	127.9	68.0
	40	195.7	141.5	75.8
K_2_SiF_6_:Mn^4+^	0	23.7	9.0	11.0
	5	45.8	28.1	20.1
	10	62.9	40.3	28.4
	15	90.3	62.5	36.4
	20	112.0	78.3	45.0
	25	133.0	93.9	53.5
	30	154.4	109.4	61.9
	35	175.0	123.7	70.5
	40	195.3	137.4	79.2

**Table 3:** Calculated shear moduli, sound velocities and Debye temperatures for the non-doped and Mn^4+^-doped K_2_SiF_6_.

System	*P*, GPa	*G*, GPa	*v_t_*, m/s	*v_l_*, m/s	*v_m_*, m/s	*Θ*_D_, K
K_2_SiF_6_	0	11.59	2146.70	3590.80	2375.61	280
	5	15.44	2278.49	4589.10	2557.08	319
	10	21.15	2547.46	5227.87	2861.95	368
	15	25.48	2708.88	5738.69	3048.37	400
	20	30.91	2909.98	6138.43	3273.96	437
	25	35.06	3037.20	6463.19	3418.59	462
	30	40.26	3199.14	6796.39	3600.57	493
	35	45.13	3331.08	7089.15	3749.39	519
	40	50.24	3467.45	7364.17	3902.49	545
K_2_SiF_6_:Mn^4+^	0	9.36	1900.48	3193.65	2104.06	248
	5	14.49	2174.34	4174.21	2432.98	303
	10	19.65	2419.06	4696.07	2708.78	348
	15	24.72	2628.23	5410.22	2953.19	388
	20	30.38	2842.32	5881.25	3194.64	426
	25	35.74	3020.83	6283.09	3396.21	459
	30	41.28	3191.09	6653.71	3588.09	491
	35	47.08	3361.47	6990.49	3779.15	522
	40	52.97	3518.48	7289.33	3954.87	551

**Table 4:** Comparison of the experimental and calculated ^2^E → ^4^A_2_ emission transition energy and 10*Dq* for the Mn^4+^-doped K_2_SiF_6_ at different pressures.

	Pressure (GPa)	Calc. (eV)	Exp. (eV)
10*Dq*	0	2.76	2.74 ^a^
	5	2.83	
	10	2.87	
	15	2.90	
	20	2.93	
	25	2.96	
	30	3.00	
	35	3.03	
	40	3.06	
^2^E → ^4^A_2_	0	2.74	1.99 ^b^
	5	2.72	
	10	2.70	
	15	2.68	
	20	2.66	
	25	2.64	
	30	2.63	
	35	2.62	
	40	2.60	

^a^ Ref. [27]. ^b^ Ref. [9].

## Data Availability

The raw/processed data required to reproduce these findings cannot be shared at this time as the data also form a part of an ongoing study.
